# Gene Expression Profiling in Human Lung Development: An Abundant Resource for Lung Adenocarcinoma Prognosis

**DOI:** 10.1371/journal.pone.0105639

**Published:** 2014-08-20

**Authors:** Lin Feng, Jiamei Wang, Bangrong Cao, Yi Zhang, Bo Wu, Xuebing Di, Wei Jiang, Ning An, Dan Lu, Suhong Gao, Yuda Zhao, Zhaoli Chen, Yousheng Mao, Yanning Gao, Deshan Zhou, Jin Jen, Xiaohong Liu, Yunping Zhang, Xia Li, Kaitai Zhang, Jie He, Shujun Cheng

**Affiliations:** 1 State Key Laboratory of Molecular Oncology, Department of Etiology and Carcinogenesis, Cancer Hospital and Institute, Peking Union Medical College and Chinese Academy of Medical Sciences, Beijing, China; 2 Department of Gynaecology and Obstetrics, Maternal & Child Health Care hospital of Haidian, Beijing, China; 3 Departments of Thoracic Surgery, Xuanwu Hospital, Capital Medical University, Beijing, China; 4 Department of Histology and Embryology, School of Basic Medical Sciences, Capital Medical University, Beijing, China; 5 College of Bioinformatics Science and Technology, Harbin Medical University, Harbin, China; 6 Departments of Thoracic Surgery, Cancer Hospital and Institute, Peking Union Medical College and Chinese Academy of Medical Sciences, Beijing, China; 7 Medical Genome Facility, and the Department of Laboratory Medicine and Pathology, Mayo Clinic. Rochester, Minnesota, United States of America; University of Barcelona, Spain

## Abstract

A tumor can be viewed as a special “organ” that undergoes aberrant and poorly regulated organogenesis. Progress in cancer prognosis and therapy might be facilitated by re-examining distinctive processes that operate during normal development, to elucidate the intrinsic features of cancer that are significantly obscured by its heterogeneity. The global gene expression signatures of 44 human lung tissues at four development stages from Asian descent and 69 lung adenocarcinoma (ADC) tissue samples from ethnic Chinese patients were profiled using microarrays. All of the genes were classified into 27 distinct groups based on their expression patterns (named as PTN1 to PTN27) during the developmental process. In lung ADC, genes whose expression levels decreased steadily during lung development (genes in PTN1) generally had their expression reactivated, while those with uniformly increasing expression levels (genes in PTN27) had their expression suppressed. The genes in PTN1 contain many n-gene signatures that are of prognostic value for lung ADC. The prognostic relevance of a 12-gene demonstrator for patient survival was characterized in five cohorts of healthy and ADC patients [ADC_CICAMS (n = 69, p = 0.007), ADC_PNAS (n = 125, p = 0.0063), ADC_GSE13213 (n = 117, p = 0.0027), ADC_GSE8894 (n = 62, p = 0.01), and ADC_NCI (n = 282, p = 0.045)] and in four groups of stage I patients [ADC_CICAMS (n = 22, p = 0.017), ADC_PNAS (n = 76, p = 0.018), ADC_GSE13213 (n = 79, p = 0.02), and ADC_qPCR (n = 62, p = 0.006)]. In conclusion, by comparison of gene expression profiles during human lung developmental process and lung ADC progression, we revealed that the genes with a uniformly decreasing expression pattern during lung development are of enormous prognostic value for lung ADC.

## Introduction

Cancer is a major public health problem. It is a leading cause of death and one in eight deaths worldwide is due to cancer [1]. A great challenge in the diagnosis and treatment of cancer arises from its ability to manifest with a great variety of pathologies and clinical behaviors due to its molecular heterogeneity. Although global gene expression profiling has helped to dissect tumor heterogeneity, e.g., breast cancer was classified into four main subtypes according to microarray data [2], this heterogeneity remains a seemingly unconquerable barrier to eliminating the uncertainties of cancer cell behavior and is a major challenge in elucidating the mechanisms of oncogenesis [3].

A tumor can be thought of as a special “organ” undergoes aberrant and poorly regulated organogenesis [4]. In contrast to oncogenesis, which is characterized by uncontrollable chaos, morphogenesis, is tightly programmed, and cell growth, death and differentiation are strictly controlled by genetic and epigenetic mechanisms. Progress may be made by re-examining distinctive processes that operate during normal development [5] to elucidate the intrinsic features of cancer that are significantly obscured by its heterogeneity.

Emerging evidence supports an intimate connection between development and oncogenesis [6]. Several studies have suggested that cancer recapitulates the gene expression patternsfound in the early developmental stages of the corresponding organ, not only for mRNAs [7], [8], [9], [10],but also for non-coding RNAs [11]. Although these findings are informative and provide novel insights into oncogenesis, those initial studies are far from perfect. First, embryonic development was studied in mice instead of humans. Because humans are evolutionarily separated from rodents by more than 70 million years [12], the mechanisms governing the development of a human embryo differ from those governing a mouse embryo, at least prior to implantation [13]. It is still unclear what other differences exist between the 3-week process of embryonic development in mice and the sophisticated 40-week process that occurs in humans. Second, although there have been analyses of gene expression profiling for the entire human embryo during early developmental stages [14], [15], none of these studies have addressed the dynamic variation of global gene expression during the development of a specific human organ or identified the common mechanisms underlying organ development and cancer progression by referencing tumor data. Third, the clinical relevance of these developmental signatures was inadequately addressed in the aforementioned studies. Although these studies provide clues for patient treatment and prognosis, the ultimate goal should have been to reveal the essence of tumor malignancy by exploring the developmental mechanisms exploited by cancer.

In this study, we compared the global expression profiles of human embryonic lung tissues and lung ADCs. It is thought that lung ADC exploits the fundamental biological mechanisms of lung development. With data from embryonic lung tissues, we identified a group of genes with a particular expression pattern during development as enriched with robust lung ADC prognostic information, which might be helpful for constructing prognosis prediction models and developing novel treatment approaches for this deadly disease.

## Materials and Methods

**Figure 1:**
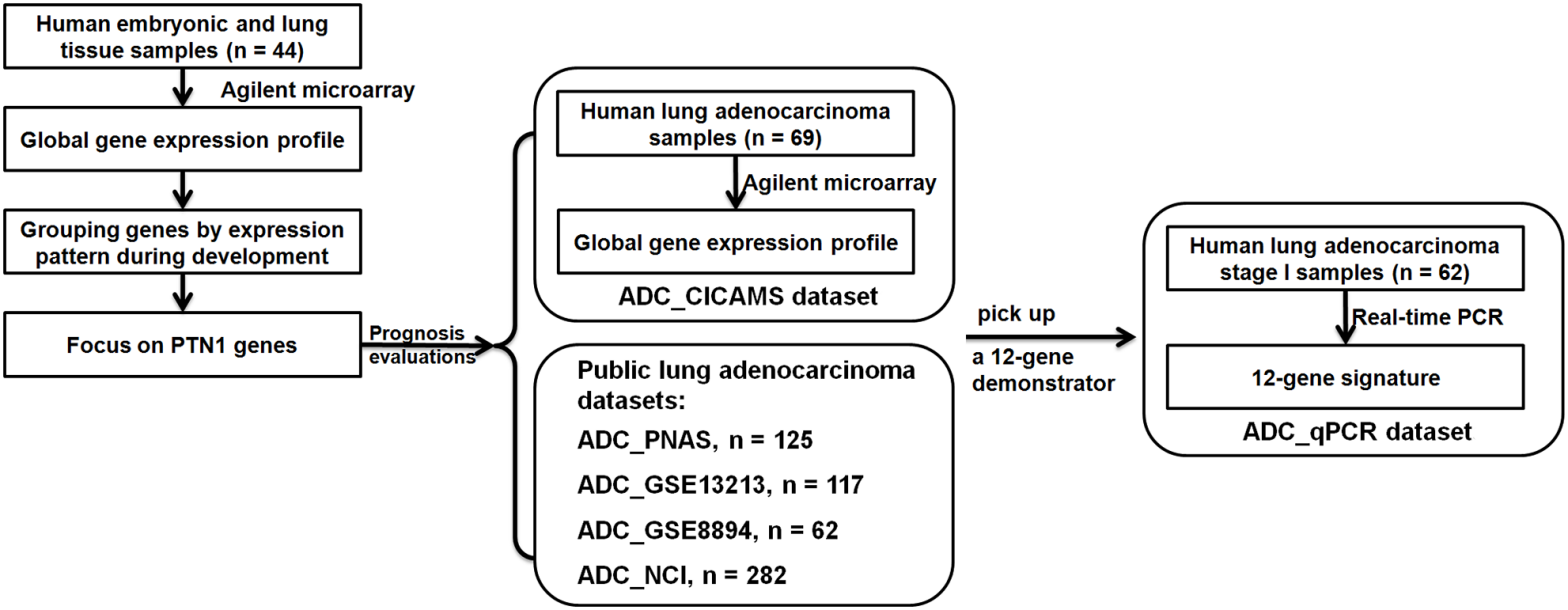
Schematic of identification and prognostic evaluation of genes with characteristic expression patterns in lung development.

A schematic for the study is depicted in Figure 1.

### Embryonic and Tumor Sample Collection

**Figure 2:**
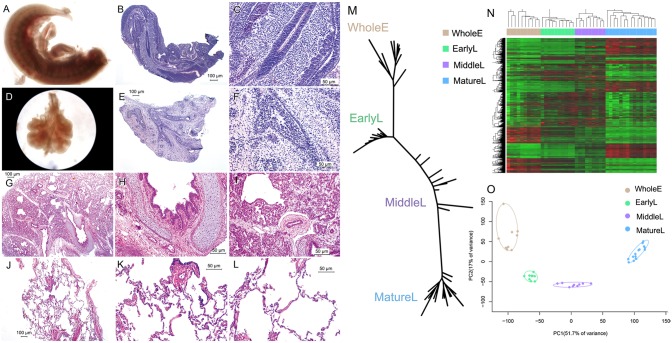
Morphological and transcriptomic features of human lung during development. (**A**–**C**), (**D**–**F**), (**G**–**I**) & (**J**–**L**), Morphological images for the four types of human developmental lung samples, i.e., WholeE, EarlyL, MiddleL & MatureL. (**M**) Cladogram was created with the whole expression profiles obtained for the developmental lung samples and shows the phylogenetic relationships among the developmental lung samples. (**N**) Hierarchical clustering analysis of top 4000 most divergent genes. For each gene, we calculated its coefficient of variation (CV) based on its expression values across all developmental samples. The genes were then ranked based on their CV values. The heatmap was generated by hierarchical clustering of the top 4000 genes with largest CV values. The colored matrix indicated the relative expression levels of genes (red for higher expression, green for lower). The distribution of samples from each developmental stage was shown above the heatmap. (**O**) Developmental samples were projected onto the two-dimensional space captured by PCA with the stages of each sample indicated by color.

The study material for the developing lung was obtained from 29 cases of spontaneous abortion at the Maternal & Child Health Care Hospital of Haidian between 2007 and 2009. The samples included whole embryos at postovulatory weeks (PWs) 3 to 5 (hereafter referred to as “WholeE”; n = 10), lungs at 6 to 8 PWs (hereafter referred to as “EarlyL”; n = 10) and at 16 to 24 PWs (hereafter referred to as “MiddleL”; n = 9). The WholeE and EarlyL samples were precisely dissected from fetal tissues with the guidance of a Nikon stereo microscope SMZ1500 (Nikon, Tokyo, Japan). MiddleL samples were collected during autopsies. Fetuses with known or suspected genetic disorders were excluded. Cancer-free peripheral lung tissue (hereafter referred to as “MatureL”) was obtained from fifteen adult patients who had undergone surgery for benign lung diseases at Xuanwu Hospital. Hematoxylin and eosin stains were used for the histological examination of the developmental samples (Figure 2). 131 lung ADC samples were obtained including 69 samples used for the expression profiling analysis (hereafter referred to as “ADC_CICAMS”) and 62 stage I samples which were used as an independent set (hereafter referred to as “ADC_qPCR”). These samples were validated by real-time PCR (qPCR) and were obtained from patients at the Cancer Institute and Hospital, Chinese Academy of Medical Sciences. The clinical features of all of the samples are presented in Table S1. All donors signed informed consent forms. The use of human tissue samples and the experimental procedures for this study were reviewed and approved by the Ethics Committee of the Cancer Institute and Hospital, Chinese Academy of Medical Sciences, and this study received the approval number 12-70/604.

### RNA Preparation

Total RNA was isolated with TRIzol reagent (Invitrogen, CA, USA). The samples allocated for microarray analysis were purified with an RNeasy kit (QIAGEN, MD, USA). The RNA was quantified by an ND-1000 UV-VIS Spectrophotometer (NanoDrop Technologies, DE, USA) and its integrity was assessed using the RNA 6000 Labchip kit in combination with the Agilent 2100 Bioanalyzer (Agilent, CA, USA). The RNA samples used in this study all exhibited OD260/280 ratios above 1.9 and RNA integrity numbers (RIN) greater than 6.5.

### Microarray Analysis

All sample-labeling, hybridization, washing and scanning steps were conducted at the Cancer Institute and Hospital, Chinese Academy of Medical Sciences, according to the manufacturer’s specifications. In brief, 1.65 µg of Cy3-labeled cRNA was generated from 500 ng of total RNA by in vitro transcription using Low RNA Input Linear Amplification Kit PLUS (Agilent) and hybridized to the Whole Human Genome Oligo Microarray (Agilent). After hybridization, the slides were washed and then scanned with the Agilent G2505B Microarray Scanner System. The fluorescence intensities on scanned images were extracted and preprocessed using Agilent Feature Extraction Software (v9.1). The raw data were normalized by the median scale method using the R package “limma” (www.r-project.org). Probes representing the same gene were further screened and only the probe exhibiting the largest mean intensity was retained. Consequently, an expression matrix containing 19,503 unique genes (listed in Table S2) was obtained and used in the subsequent analysis. The raw and processed data are publicly available on the Gene Expression Omnibus (GEO) website under the accession number GSE43767.

### Real-time PCR assays

Two µg of RNA was converted to cDNA using Superscript II (Invitrogen, CA, USA) in accordance with the manufacture’s protocol for a final volume of 20 µl. The TaqMan method was employed for the qPCR analysis of 13 genes (including 12 target genes and one reference gene *POLR2A* [16], Table S3). We performed qPCR analysis on the Mx3005P QPCR System (Agilent) using the TaqMan Gene Expression Assays kit (Applied Biosystems, CA, USA). Relative mRNA expression was calculated using the comparative Ct method, and a greater ΔCt corresponds to a lower gene expression level.

### Analysis of Public Microarray Datasets

Four independent sets of lung ADC microarray data (“ADC_GSE13213” [17], “ADC_GSE8894” [18], “ADC_PNAS” [19] and “ADC_NCI” [20]) and their corresponding clinical information (Table S1) were collected from existing publications for validation. The raw data from ADC_GSE13213, ADC_PNAS and ADC_NCI were normalized using the same method used for the ADC_CICAMS group. Because the raw data for ADC_GSE889 were not provide, GCRMA processed data were downloaded and analyzed directly.

### Grouping Genes by Expression Pattern during Development

**Figure 3:**
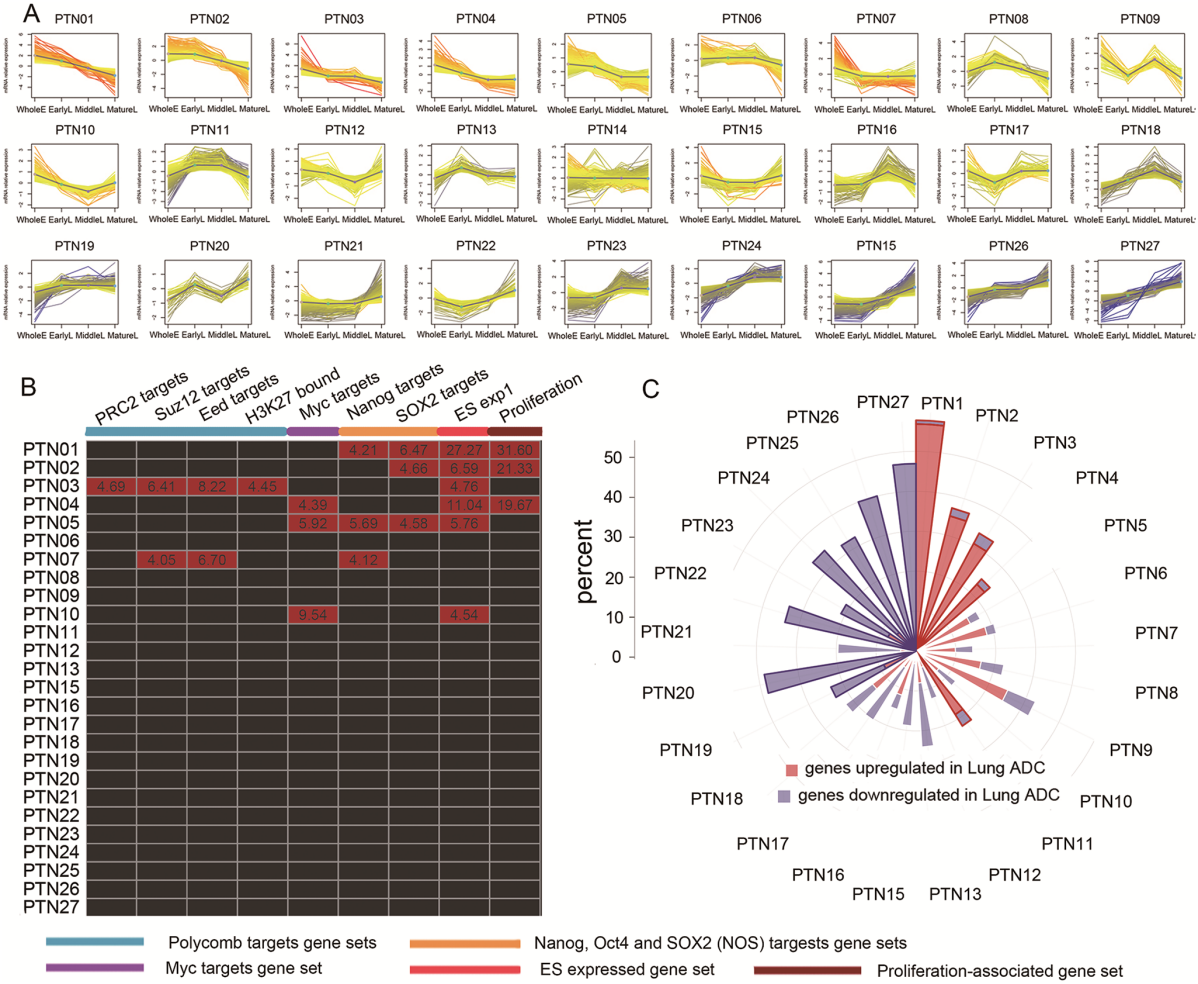
Global gene classification and functional annotation. (**A**) The genes were classified into 27 PTNs according to their expression dynamics throughout the lung development process. The time points during development were plotted on the x-axis, and the normalized gene expression level in every panel was plotted on the y-axis. Each gene is depicted with a line colored according to its relative expression level at the corresponding time points. (**B**) The results of the gene set enrichment analysis of vPTNs and ES-related gene lists are indicated by the color of the corresponding box, with red representing significant enrichment (the number in the red box indicates the negative log10 of the enrichment p value) and black representing the absence of enrichment. (**C**) The rose diagram displays the distribution of lung ADC-related genes in 26 vPTNs. The proportions of lung ADC-related genes in vPTNs are represented by the length of the petals, with red and blue indicating up- and downregulated genes, respectively. The rose petals corresponding to vPTNs significantly enriched with genes that were up- and downregulated in ADC are highlighted by colored outlines while those corresponding to vPTNs without enrichment are outlined in white.

The tissue samples were divided into the following four developmental stages: WholeE, EarlyL, MiddleL and MatureL. Gene expression patterns during lung development were defined by the manner in which the expression levels changed relative to those changes found in neighboring stages. In general, the unpaired Student’s t-test was applied to identify differentially expressed genes for each pair of adjacent time points (p<0.05 and FDR<0.01 was used as a significance level). There are three scenarios for differences in gene expression between successive time points: upregulation (u), downregulation (d), and no significant change (n). Accordingly, the genes can be divided into 27 (the number of permutations of three elements chosen from “u”, “d” and “n” allowing for repetition) groups defined by the three transitions among four stages (Figure 3 and Table S2). The group in which gene expression decreased steadily as lung development occurred, i.e., “uuu”, is hereafter referred to as “PTN1”. The group consisting of genes with no significant change in expression levels over the four developmental stages (“nnn”) is referred to as “PTN14”. The other patterns were sorted according to their Pearson’s correlation coefficients relative to “PTN1” and designated sequentially from “PTN2” to “PTN27” with the exclusion of “PTN14”.

### Prognostic Signature Permutation

To further investigate the relationship between the genes with characteristic expression patterns during development and the clinical phenotypes of lung ADC patients, a permutation test for prognostic signatures was conducted for genes in PTN1 using our ADC_CICAMS and the four existing lung ADC microarray datasets (i.e., “ADC_GSE13213”, “ADC_GSE8894”, “ADC_PNAS” and “ADC_NCI”), for which the flowchart was shown in Figure S1. In brief, firstly, n (n = 3, 6, 9, …, 21) different genes were randomly chosen from a given gene list (see below for details) to consist of a n-gene signature (where n represents the number of genes constituting the signature). Secondly, k-means clustering (k = 2) was performed to divide patients in each dataset into two groups based on the expression level of the n-gene signature. Then Kaplan-Meier estimates of the overall survival in the two groups of patients were performed, and only those signatures that correlated significantly (log rank test p<0.05) with the prognosis of lung ADC patients in all five datasets were defined as robust effective signatures. This procedure was repeated 10,000 times for any given n and for each of two gene lists, i.e., PTN1 and a list containing 200 fixed genes randomly chosen from the global genes expression profile (hereafter referred to as Random200). For the two gene lists, the fraction of robust effective signatures among the 10,000 signatures was calculated.

### Statistical Analysis

The gene sets annotation were done with DAVID tools (http://david.abcc.ncifcrf.gov/). Gene set enrichment was analyzed using Genomica software [21] with a significance level of α = 0.0001. The rest statistical analyses of this work were all conducted with R software. In details, the phylogenetic analysis, principal component analysis (PCA) and the unsupervised hierarchical clustering analysis were carried out with the R package “ape” and “stat” package, respectively. The lung ADC-related genes were defined as the genes differentially expressed between the lung ADC and MatureL groups and were identified using an unpaired Student’s t test (R package “stat”) with a Bonferroni correction adjusted p value of 0.01, which was accepted as the significance level. The sampling scheme without replacement (from R package “base”) was used to choose 10,000 n-gene signatures. Correlation between the n-gene signatures and lung ADC patient prognosis was evaluated by the log rank test (R package “survival”) with the patients stratified by the signatures.

## Results

### Morphologic and Transcriptomic Features of Human Lung Morphogenesis

Human embryonic and lung tissue samples were collected from four developmental stages (i.e., WholeE, EarlyL, MiddleL, and MatureL) and morphologically examined. The samples apparently underwent a continuous and sequential maturation process and diverse cell types gradually appeared. Basic tissue architecture was starting to emerge in WholeE (Figure 2A–C) samples: the embryo was C-shaped and somites had appeared. The EarlyL group (Figure 2D–F) exhibited a few tubulo-acinous gland-like structures mainly composing epithelial buds or atypical adenoid structures. A large number of stromal cells filled the space between the bronchia. Blood vessels, hyaline cartilage and smooth muscle were found in the wall of the bronchi in the MiddleL group (Figure 2G–I). The lumina were initially coated with cubic epithelial cells, and a portion of the epithelial cells gradually changed into thin, flat cells as the residual stromal cells around the lumina were reduced. During the mature stage (MatureL, Figure 2J–L), alveolar ducts were lined with a simple epithelium supported by smooth muscle fibers, and the pulmonary alveoli exhibited very thin walls lined with flattened pneumocytes.

In addition to the continuous changes observed during the morphological analysis, phylogenetic analysis, unsupervised cluster and PCA all indicated that the transcriptomic features of lung ontogenesis are arranged in a sequential order according to the time of development (Figure 2M–O). Samples clustered tightly within each developmental stage, whereas the different stages were distinctly separate. The gestation ages of the MiddleL samples widely varied (spread over an 8 weeks period); therefore, this group of samples was loosely dispersed on the trunk of the phylogenetic tree, in contrast to the other three groups of samples, which constituted respective independent “branches”. As inferred from this cladogram, samples obtained from each stage possessed very distinct molecular profiles leading to the manifestation of different morphological features.

### Identification of Genes with Characteristic Expression Patterns in Human Lung Development

The genes were divided into 27 groups (named as PTNs) according to their expression level dynamics during lung development (Table S2 and Figure 3A). The largest group, PTN14, contained more than 5,000 genes that showed no significant changes during development and was therefore not involved in the subsequent analysis. The other 26 PTNs were hereafter referred to as vPTNs, among which genes in PTN1 and PTN27 showed similar changing trends of either decreased or increased expression levels as development progressed.

Gene set enrichment analysis of vPTNs was performed to identify shared biological themes using DAVID Bioinformatics Resources (Table 1). Genes in small-numbered PTNs were related to proliferation, except for PTN3 which was enriched with genes mediating differentiation. In contrast, genes in large-numbered PTNs were commonly involved in cell-cell communication, interaction with the extracellular matrix, apoptosis or other biological processes related to immune response.

**Table 1:** The most significantly enriched functional categories and GO terms of genes in each vPTN with corresponding enrichment score (ES).

Patterns	Genes No.	Functional Categories	ES	Gene Ontology (BP)	ES
PTN01	213	cell cycle/mitosis	28.81	cell cycle	30.60
PTN02	634	cell cycle/mitosis	24.99	cell cycle	22.04
PTN03	239	dna-binding/transcriptionregulation	5.34	development/differentiation	6.58
PTN04	521	cell cycle/mitosis	8.47	cell cycle	11.35
PTN05	1068	mitochondrion	14.27	RNA splicing	16.14
PTN06	1817	zinc-finger/transcriptionregulation	18.65	regulation of transcription	16.39
PTN07	1011	dna-binding/transcriptionregulation	5.18	pattern specification process	4.12
PTN08	175	zinc-finger/transcriptionregulation	1.55	regulation of transcription	1.79
PTN09	40	chromosomalprotein/dna-binding/acetylation/methylation	1.23	DNA packaging	1.75
PTN10	305	mrna processing/mrnasplicing	5.73	RNA splicing	5.82
PTN11	920	zinc-finger/transcriptionregulation	4.56	regulation of transcription	4.98
PTN12	421	respiratory chain/electrontransport	4.05	protein catabolic process	4.43
PTN13	137	Secreted/signal/glycoprotein	1.73	bone development	1.51
PTN15	1081	nuclear pore complexm/RNAtransport/translocation	2.34	protein localization	2.93
PTN16	241	cilium/cell projection	2.06	cell motility	1.46
PTN17	123	cilium/cytoskeleton	1.48	determination of bilateralsymmetry	1.75
PTN18	95	domain:Fibronectin type-III	1.08	gamete generation	0.80
PTN19	661	integrin	1.65	angiogenesis	2.23
PTN20	39	Immunoglobulin domain	1.13	negative regulation ofmacromolecule metabolicprocess	0.52
PTN21	2440	ribosome	16.82	vesicle-mediated transport	5.75
PTN22	116	sodium/potassium transport	1.37	regulation of lipid metabolicprocess	1.57
PTN23	586	cilium/cytoskeleton	4.75	protein amino acidphosphorylation	2.02
PTN24	191	Secreted/signal/glycoprotein	2.73	positive regulation oftranscription	3.09
PTN25	646	Secreted/signal/glycoprotein	12.11	defense response	14.04
PTN26	270	Secreted/signal/glycoprotein	2.61	activation of immuneresponse	3.09
PTN27	209	Secreted/signal/glycoprotein	4.32	apoptosis	4.70

Furthermore, we compared vPTNs with gene sets reflecting embryonic stem (ES) cell identity as reported in Ben-Porath et al. [22]. The original gene sets including the ES expressed ES exp1, *Nanog*, *Oct4*, and *SOX2* (NOS) targets, Polycomb targets, and *Myc* targets, and a proliferation-associated gene set was also obtained. We focused on 9 out of the 13 ES related gene sets and the proliferation-associated gene set described by Ben-Porath. As shown in Figure 3B, these gene sets were significantly enriched in small-numbered groups, e.g., PTN1 to 5. As expected, PTN1, PTN2 and PTN4, which were identified as proliferation-related genes by GO analysis, were also enriched in the proliferation-associated gene set and are thought to play a role in stem cell self-renewal. In contrast, PTN3 was involved in differentiation and was enriched for Polycomb targets, the four sets comprising genes bound by the Polycomb repressive complex 2[22] which is proven to be the molecule essential for stem cell maintenance [23] and differentiation [24]. This analysis indicates that the genes found in these patterns might reveal the core stem cell properties (or “stemness”, which refers to the ability of a cell undergo self-renewal and generate differentiated progeny), and reflect the developmental potential of these samples. PTN15 to 27 failed to attain the enrichment significance level required for enrichment.

### Association between Lung Development and Adenocarcinoma Progression at Transcriptome Level

Firstly, we examined whether genes related to lung ADC showed particular expression patterns during lung development. Gene expression profiles from 69 lung ADC samples were generated by microarray analysis (ADC_CICAMS). We found that 2,121 genes exhibited upregulated expression in lung ADCs and 1,688 genes were downregulated (Table S2). Distribution of these ADC-related genes into the 26 classes of vPTNs is shown in a rose diagram (Figure 3C). As the PTN number increased, the proportion of genes in each vPTN upregulated in ADC gradually decreased, whereas the proportion of those genes downregulated in ADC gradually increased. This trend can be seen in the two-colored rose diagram, in which up- and downregulated genes are indicated in red and blue, respectively; red genes are concentrated on the right, and blue genes are concentrated on the left. Statistical analysis showed that the up- and downregulated ADC-related genes were significantly enriched in small- and large-numbered PTNs, respectively.

**Figure 4:**
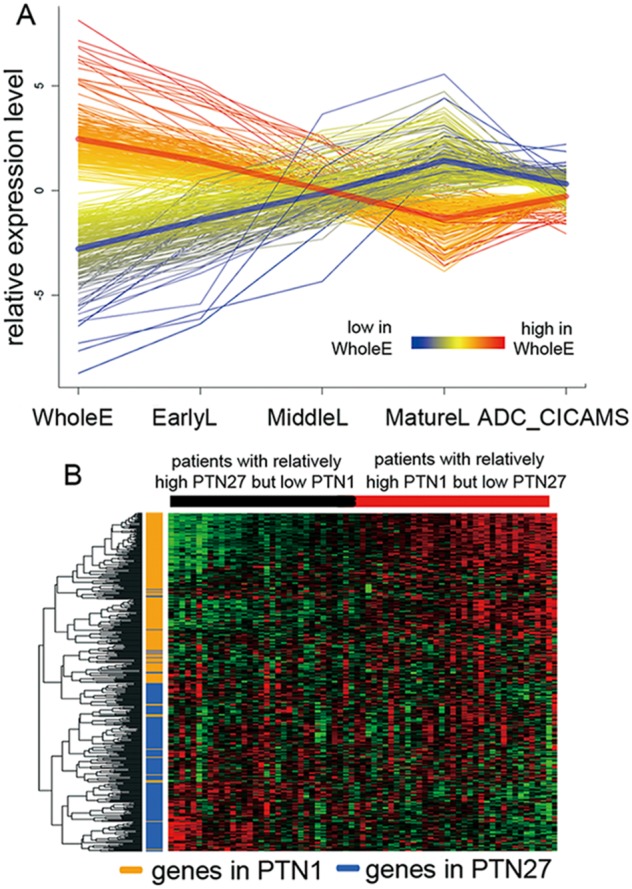
The antagonistic relationship of genes in the PTN1 and PTN27 group. (**A**) Genes in PTN1 and PTN27 were represented by lines as in Figure 3A, except for the right-most section which is the extension of the corresponding gene’s expression level in lung ADC. The average expression level of each gene of the pattern is represented by a thick red line for PTN1 and a blue line for PTN27. (**B**) Hierarchical clustering of genes in PTN1 and PTN27 from the ADC_CICAMS dataset. The expression levels of PTN1 and PTN27 genes are illustrated as a color spectrum, with red, black and green representing high, medium and low expression, respectively, in a matrix indexed by genes in rows and samples in columns. The genes were specified on the left side of the matrix by short lines colored orange for PTN1 or blue for PTN27.

Next, to more explicitly examine the reinstatement of lung developmental related genes in the process of ADC progression, genes in PTN1 and PTN27 representing the two diametrical extremes with the most significant monotone contrast were intensively analyzed. Figure 4A shows the average expression levels of these genes during the four developmental stages examined and in lung ADC samples. It is clear that during ADC tumorigenesis, genes progressively repressed during development generally reactivated their expression, whereas those with steadily increasing expression were suppressed. Although overexpressed PTN1 and repressed PTN27 genes were common in tumor tissues, the degree of this phenomenon varied among tumor tissues obtained from different patients. According to the results of the hierarchical clustering analysis (Figure 4B), these 422 genes were grouped into two major clusters based on their expression correlation among different ADC samples (ADC_CICAMS). The clustering outcome indicated that the genes in PTN1 and PTN27 were neatly separated revealing that the correlation status of the corresponding genes in the PTN1 and PTN27 groups still holds in lung ADCs. Moreover, the expression patterns of these genes in lung ADC tissues were related to their clinical phenotypes. Patients with tumor tissues showing higher expression of PTN1 genes and repressed expression of PTN27 genes have higher TNM stages (Chi-square test, p = 0.002), poorer differentiation of tumors (Chi-square test, p = 0.005) and worse prognosis (i.e., more died of cancer within 3 years after surgical operation) (Chi-square test, p = 0.009). Other clinical parameters such as gender, age, smoking index and T stage were not significant.

### Prognostic Significance of PTN1 Genes for Lung ADC Patients

**Figure 5:**
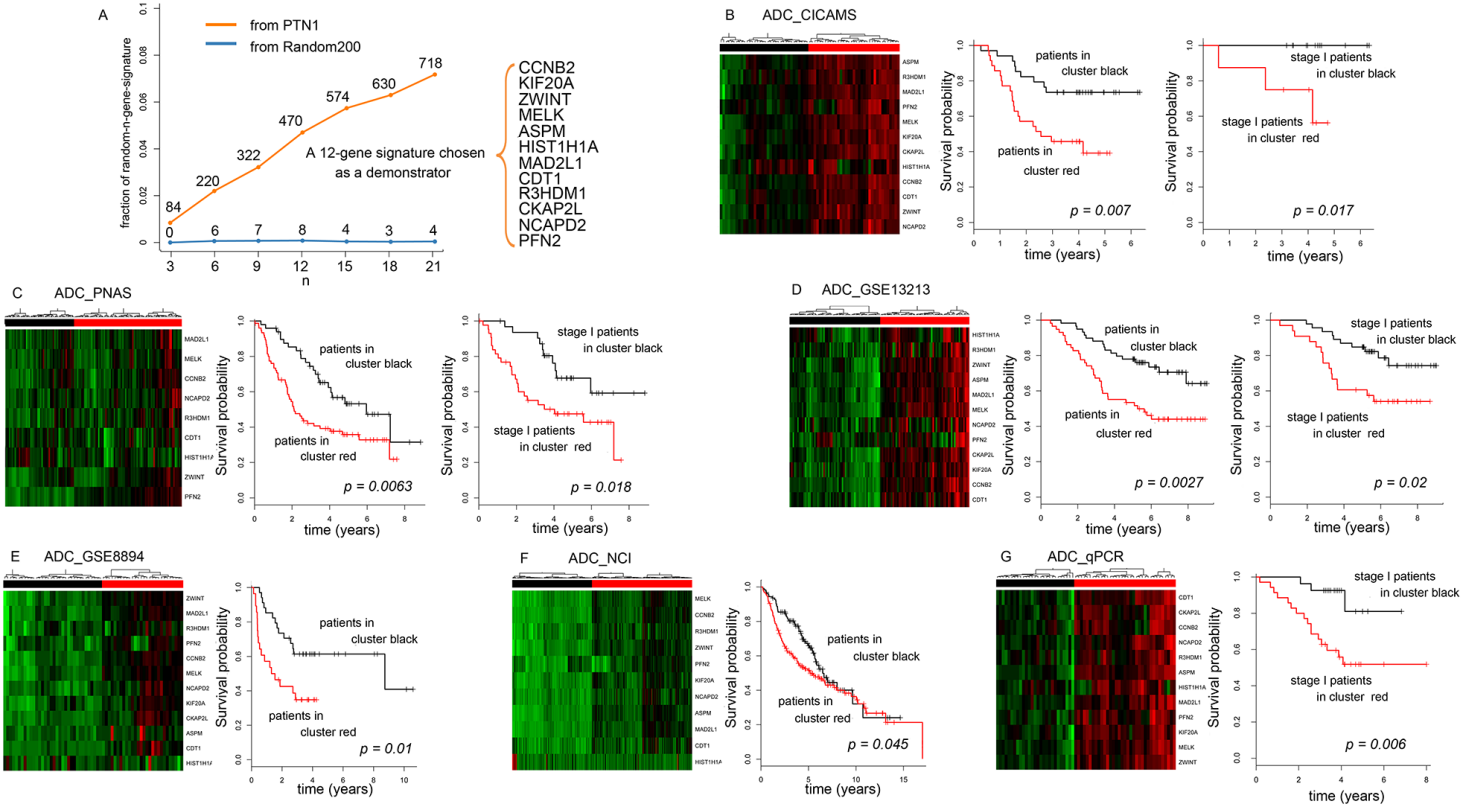
The prognostic value of PTN1 genes for lung ADC patients. (A) The proportion of random-n-gene signatures associated with prognosis in all five groups of lung ADC patients. The number labeled along the two lines indicates how many n-gene signatures selected from the corresponding gene list were related to the prognosis of all five groups of lung ADC patients at 10,000 sampling times. (B–G) Survival analysis of six groups of independent lung ADC patients stratified by a representative random 12-gene signature. Patients were classified into two major groups (left panel) by unsupervised hierarchical clustering. The colored matrix indicates the relative expression levels of genes (red for higher expression, green for lower). Kaplan-Meier survival curves and log-rank tests were used to estimate survival in the five lung ADC microarray datasets for patients at all disease stages (B–D middle panel, and E–F right panel) for the three groups of stage I patients (B–D right panel) and for an independent group of stage I lung ADC patients (G right panel, qPCR data).

To further investigate the relationship between genes with characteristic expression patterns during development and the clinical phenotypes of lung ADC patients, 10,000 n-gene signatures (n = 3, 6, 9, …, 21) were randomly selected from the PTN1 and Random200 (200 genes randomly chosen from global gene expression profile) groups and were examined for their prognostic significance in ADC_CICAMS and four existing lung ADC microarray datasets. Survival analysis revealed that PTN1 contains a much higher proportion of random n-gene signatures prognosis-associated for all five patient groups (Figure 5A). It implies that the genes in PTN1 may be valuable for lung ADC patient prognosis. To illustrate the correlation between PTN1 genes and patient prognosis as well as the robustness thereof, we selected one of the 470 12-gene signatures (out of 10,000 12-gene signatures) that were related to the prognosis of all five lung ADC patient groups. As shown in Figure 5B–F, the left panels indicate five groups of patients stratified by unsupervised hierarchical clustering according to the expression level of the 12-gene signature. The log-rank test results indicate a significant difference in prognosis between high expression (red) and low expression (black) clusters (ADC_CICAMS, n = 69, p = 0.007; ADC_PNAS, n = 125, p = 0.0063; ADC_GSE13213, n = 117, p = 0.0027; ADC_GSE8894, n = 62, p = 0.01; ADC_NCI, n = 282, p = 0.045). Furthermore, expression level of the signature in tumor tissues was significantly associated with overall survival in three independent groups of stage I lung ADC (right panel of Figure 5B–D, log-rank test: ADC_CICAMS, n = 22, p = 0.017; ADC_PNAS, n = 76, p = 0.018; ADC_GSE13213, n = 79, p = 0.02).

To validate the prognostic potential of this 12-gene signature, we analyzed 62 independent ADC stage I samples by qPCR. As shown in Figure 5G, survival analysis revealed a significant difference between the clinical outcomes of the two groups identified by hierarchical clustering (log-rank test, p = 0.006), with the group showing a high expression level (low ΔCT value) corresponding to a worse prognosis similar to the results obtained from the microarray analysis.

## Discussion

A new research approach is emerging that examines embryonic development for information regarding the malignant transformation of tumor cells [25]. For the first time, the gene expression profiles of human lung development were described in this study, a large amount of fundamental data are provided for future research, and gene expression patterns in conjunction with their underlying biological functions were analyzed. Subsequently, the dynamics of gene expression were compared in lung development and lung ADCs, thereby demonstrating that primary lung ADC may partially exploit the molecular mechanisms governing lung development by down-regulating the expression of the PTN27 genes and up-regulating the expression of the PTN1 genes. The genes in PTN1 with a steadily decreasing expression pattern during lung development were enriched with information valuable for lung ADC prognosis. In addition, the relationship between PTN1 genes and lung ADC prognosis is very robust. While it can be argued that the gene signature revealed in our study was a result of statistical chance observation, the relationship between PTN1 genes and the similar prognostic prediction among six independent groups of lung ADC patients, including one group from which data were analyzed by qPCR, strongly support the highly robustness of the signature and the potential for their use in clinical settings. Particularly given the observation that this relationship is independent of the patients’ clinical stage, being fairly significant even in stage I patients.

Predicting the prognosis of cancer is a major challenge in current clinical research [26]. During the last decade, numerous gene lists were derived from global gene expression data and reported to be prognostic for cancer patients [27], [28], [29], [30]. Simultaneously, the reliability of the contents of these gene lists and their predictive value have been widely discussed [31], [32], [33]. After all, the predictive value of the signatures depends on the strength and robustness of the candidate genes used to build the model, regardless of the improvement in methods for statistics and model construction [34], [35]. In this study, we revealed that genes with a steadily decreasing expression levels over the course of lung development contain lung ADC prognostic information and that PTN1 might be a good knowledge-based candidate list for researchers focused on constructing prognosis predictors for lung ADC patients.

This study found that the genes in PTN1 are associated with cell proliferation, which is consistent with published evidence that proliferation may underlie the prognostic power of many previously identified signatures [36], [37], [38] and which may partially explain why PTN1 genes are so powerful in terms of prognostic association. PTN1 is also enriched in genes highly expressed in ES cells, especially those regulated by *Nanog* and *Sox2*, pointing to the likelihood that the expression level of PTN1 genes in lung ADC tissue might reflect the aggressiveness of the cancer, which is one potential factor contributing to disease recurrence [39]. This might be another reason for the prognostic significance of the PTN1 genes. Furthermore, the genes in PTN1 exhibit quite a similar expression pattern during lung development, suggesting that they are regulated by one or several mechanisms. Identifying the functional regulators of these genes and designing relevant drugs may facilitate the discovery of a new target for cancer treatment. It is our hope and expectation that the study of lung development will enable us to better understand cancer pathogenesis and ultimately improve therapeutics.

## Supporting Information

Figure S1 **Flowchart of prognostic signature permutation.** The cartoon depicted the process of prognostic signature permutation.
(PDF)

Table S1 **The clinical information of all lung ADC patients involved in this study.**
(XLSX)

Table S2 **The global genes divided into 27 PTNs.**
(XLSX)

Table S3 **The gene symbol and ABI assay ID of 12-gene signature and reference gene.**
(DOCX)
