# Supplementary material for: Gene Expression Profiling in Human Lung Development: An Abundant Resource for Lung Adenocarcinoma Prognosis
Source: PLoS One. 2014 Aug 20;9(8):e105639. doi: 10.1371/journal.pone.0105639 (PMC4139381; doi:10.1371/journal.pone.0105639)
Supplement: Figure S1 — Flowchart of prognostic signature permutation. The cartoon depicted the process of prognostic signature permutation. (PDF) [file pone.0105639.s001.pdf]

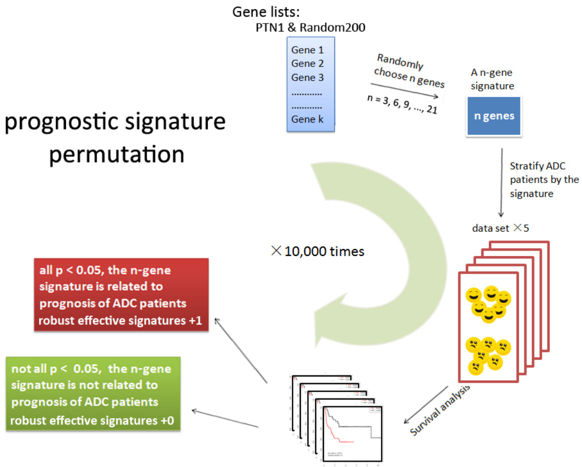
Gene lists:
PTN1 & Random200
Gene 1
Gene 2
Gene 3
....
....
Gene k
Randomly choose n genes
n = 3, 6, 9, ..., 21
A n-gene signature
n genes
prognostic signature permutation
Stratify ADC patients by the signature
data set ×5
×10,000 times
all p < 0.05, the n-gene signature is related to prognosis of ADC patients
robust effective signatures +1
not all p < 0.05, the n-gene signature is not related to prognosis of ADC patients
robust effective signatures +0
Survival analysis
