# Supplementary material for: Gene Expression Profiling in Human Lung Development: An Abundant Resource for Lung Adenocarcinoma Prognosis
Source: PLoS One. 2014 Aug 20;9(8):e105639. doi: 10.1371/journal.pone.0105639 (PMC4139381; doi:10.1371/journal.pone.0105639)
Supplement: Table S3 — The gene symbol and ABI assay ID of 12-gene signature and reference gene. (DOCX) [file pone.0105639.s004.docx]

Supplementary Table 2. The gene symbol and ABI assay ID of 12-gene signature and reference gene.

| Gene Symbol | ABI assay ID |
| --- | --- |
| CCNB2 | Hs01084593_g1 |
| KIF20A | Hs00993573_m1 |
| ZWINT | Hs01037853_g1 |
| MELK | Hs01106440_m1 |
| ASPM | Hs00270424_m1 |
| HIST1H1A | Hs00271225_s1 |
| MAD2L1 | Hs03063324_g1 |
| CDT1 | Hs00368864_m1 |
| R3HDM1 | Hs00990838_m1 |
| CKAP2L | Hs00967782_m1 |
| NCAPD2 | Hs01027658_g1 |
| PFN2 | Hs00975963_m1 |
| POLR2A (reference gene) | Hs01108284_g1 |
